# Axillary Nodal Metastases from Carcinoma of Unknown Primary (CUPAx): Role of Contrast-Enhanced Spectral Mammography (CESM) in Detecting Occult Breast Cancer

**DOI:** 10.3390/jpm11060465

**Published:** 2021-05-25

**Authors:** Melania Costantini, Rino Aldo Montella, Maria Paola Fadda, Giorgia Garganese, Alba Di Leone, Alejandro Martin Sanchez, Gianluca Franceschini, Pierluigi Maria Rinaldi

**Affiliations:** 1 Radiology Unit, Mater Olbia Hospital (Qatar Foundation Endowment and Policlinico Universitario Agostino Gemelli IRCCS Foundation), 07026 Olbia, Italy; melania.costantini@materolbia.com (M.C.); mariapaola.fadda@materolbia.com (M.P.F.); pierluigi.rinaldi@materolbia.com (P.M.R.); 2 Area Diagnostica per Immagini, Dipartimento Diagnostica per Immagini, Radioterapia Oncologica ed Ematologia, Fondazione Policlinico Universitario Agostino Gemelli IRCCS, 00168 Roma, Italy; 3 Gynecology and Breast Care Center, Mater Olbia Hospital (Qatar Foundation Endowment and Policlinico Universitario Agostino Gemelli IRCCS Foundation), 07026 Olbia, Italy; giorgia.garganese@materolbia.com; 4 Dipartimento Scienze della Vita e Sanità Pubblica, Sezione Ginecologia e Ostetricia, Università Cattolica del Sacro Cuore, 00168 Roma, Italy; 5 Multidisciplinary Breast Center, Dipartimento Scienze della Salute della Donna e del Bambino e di Sanità Pubblica, Fondazione Policlinico Universitario Agostino Gemelli IRCCS, 00168 Roma, Italy; alba.dileone@policlinicogemelli.it (A.D.L.); martin.sanchez@policlinicogemelli.it (A.M.S.); gianluca.franceschini@policlinicogemelli.it (G.F.); 6 Istituto di Semeiotica Chirurgica, Università Cattolica del Sacro Cuore, 00168 Roma, Italy

**Keywords:** CUP syndrome, axillary lymph node metastases, occult breast cancer, contrast-enhanced spectral mammography, breast imaging

## Abstract

Axillary lymph node metastases of occult breast cancer (CUPAx) is an unusual condition that represents both a diagnostic and therapeutic challenge. The first steps in the diagnostic work-up of patients with CUPAx are the histological analysis of the lymph node metastasis and the execution of basic breast diagnostic imaging (mammography and ultrasound). In the case of occult breast cancer, breast Magnetic Resonance (MR) must be performed. Breast MR identifies a suspicious lesion in many patients and second-look ultrasound detects a corresponding ultrasound alteration in about half of cases, allowing the performance of a US-guided biopsy. In the case of an MR-only lesion, MR-guided biopsy is mandatory. We present a case of CUPAx in which contrast-enhanced spectral mammography (CESM) is used to help the detection of occult breast cancer and to guide stereotactic vacuum breast biopsy (VABB). CESM is a new breast imaging technique that is proving to have good performance in breast cancer detection and that is showing potential in the identification of occult breast cancer in a CUPAx setting. The use of an innovative and personalized breast imaging approach in breast cancer patients improves diagnostic possibilities and promises to become the focus in decision strategies.

## 1. Introduction

The term CUP (cancer of unknown primary) syndrome identifies a metastatic disease of a primitive occult cancer that remains unknown after a comprehensive diagnostic work-up. CUP syndrome is a highly aggressive disease with early metastatic dissemination, accounting for approximately 2–3% of all diagnosed cancer [1,2,3,4,5].

Very early primary cancer or stem cells with metastatic potential are probably the triggers of the syndrome [6,7].

Although the acronym CUP refers to a heterogeneous group of tumors, patients generally have a short and non-specific clinical history and unfavorable prognosis. The incidence is highest in men over the age of 60 [1,2,4,8].

Most patients with CUP syndrome have epithelial-type tumors, especially adenocarcinoma or poorly differentiated carcinoma. Only in a small percentage of cases the tumors are of mesenchymal origin [1,4,6].

More than 50% of patients with CUP syndrome have disseminated metastases, and the remaining ones have a single site involvement such as the liver, bones, lung or nodes. Only 15% of patients have a singular nodal site involvement [1,4,8].

When a metastatic disease is diagnosed in the absence of a primary cancer site, careful selection of diagnostic tests is mandatory to maximize the possibility of identifying the primary lesion and accessory metastatic sites, to minimize the delay in starting therapeutic treatment and to reduce the risk for the patient.

According to the international guidelines, in order to localize a potential primary tumor, the initial diagnostic algorithm includes a detailed medical history, a complete physical examination, the needed laboratory tests and a whole body dynamic computed tomography (CT) and/or a whole body positron emission tomography–computed tomography (PET/CT) [4,6,9,10].

Patients who performed selected initial tests based on cytology and histology that showed metastatic malignancy without any primary site of origin belong to the “provisional CUP” group. Histological evaluation of the metastatic lesion and specific pathology investigation (immunohistochemistry and molecular diagnosis) are essential to guide any additional diagnostic process [4,8]. Recently, advances in genomic profiling offer new potential to identify the origin cancer tissue [1,3,6,11].

Furthermore, “provisional CUP” becomes “confirmed CUP” if more additional specialist evaluations and specialized investigations (organ-specific) have been performed and the primary site remains unknown. Therefore, CUP syndrome is a diagnosis of exclusion and this algorithm helps to differentiate patients in different phases of the diagnosis process [1,4,6,10,12]. Patients with confirmed CUP are treated by empiric chemotherapy, assuming that primary tumors have an epithelial derivation. However, overall survival still remains very poor, with a median duration of less than one year. Today, the only strategy to improve the outcome of this disease is the identification of the primary specific cancer type in order to use a more precise targeted therapy [1,6,7,13].

The understanding of the biologic behavior of these tumors allowed the recognition of two groups of patients based on prognosis. Patients in the favorable subset, approximately 20% of all CUP, are responsive to systemic chemotherapy and/or locoregional treatment because a presumptive occult primary site has been assumed. Approximately 80% of other CUP patients belong to the non-favorable subset because the origin tumor remains unknown and the response to empirical treatments is very poor [2,3,13].

Female patients with axillary lymph node metastases (CUPAx) of occult primary are considered a favorable subset. The most common cause of metastatic axillary lymph nodes is ipsilateral breast cancer. However, other adenocarcinomas can metastasize to the axilla, in particular lung, thyroid, stomach, colorectal and pancreatic cancers. Therefore, patients with axillary lymph node metastases of an unknown primary represent an important diagnostic dilemma for the clinician [4,8,14].

In 0.3–1% of all women with breast cancer, metastatic adenopathy of the axilla is the first symptom of the disease and it occurs mainly in the post-menopausal age. It is suggested that around two-thirds of these patients will have an occult breast primary within the ipsilateral breast [10,15,16].

Nowadays, few data concerning the specific clinical patterns of CUPAx female patients are available in the literature. A recent manuscript reviewed patients’ characteristics, showing only a predominance of a favorable molecular receptor profile and a higher histological grade [17].

The fulcrum of the diagnostic strategy is the biopsy of the pathological lymph node and the immunohistochemical and molecular analyses of the sample. When the histology confirms an adenocarcinoma or a poorly differentiated carcinoma, the presence of occult ipsilateral breast cancer is the most likely [10].

Mammography and breast ultrasound (US) are the first imaging modalities to detect primary occult breast cancer [10,14,15].

The next step is to perform magnetic resonance imaging of the breast [18,19]. Breast MR is the most accurate method for detecting breast cancer, with almost 100% sensitivity and around 70% specificity. The not-very-high specificity highlights the problem in managing the false positives and the consequent unnecessary biopsies. The main strategies to bypass this intrinsic limitation are represented by the use of high-performance machines and coils, adequate protocols and strict compliance with the indications established by the current guidelines [18,19].

Breast MR is a consolidated indication for searching for a primary occult breast tumor in CUP syndrome when first-level examinations did not reveal suspicious lesions. Breast MR now correctly identifies the primary cancer in more than half of the population. MR not only has the potential to identify the suspected lesion but also plays an important role in tumor staging [18,19].

In the case of an MR-only lesion, MR-guided breast biopsy represents the only way to detect occult breast cancer [20].

Recently, CESM has shown potential to increase the diagnostic possibilities of mammography, suggesting its use in various diagnostic challenges [21,22].

CESM is a special type of mammogram that uses a dual energy exposure in combination with intravenous contrast medium administration. For each mammogram projection, a pair of low- and high-energy images are acquired and the relative subtracted images are obtained [21,22].

Therefore, CESM offers both a regular mammogram image and a subtracted image that contains dynamic information. CESM highlights areas of abnormal blood flow supply in the breast, helping the detection of tumor neovascularity similar to breast MR imaging.

We present a case of CUPAx in which a personalized breast imaging approach with the use of CESM allowed the detection of occult breast cancer.

## 2. Case Presentation

We report the case of a CUPAx syndrome in which CESM played a key role in the correct management.

A 30-year-old Caucasian female patient with a history of cigarette smoking with no comorbidity underwent mammography and breast ultrasound because of palpable mass in the left axillary region.

Clinical familiar history was negative for cancer cases.

Breast ultrasound demonstrated some enlarged and suspicious axillary nodes (largest node dimension: 45 mm) and a small low-suspicious hypoechoic area in the retro-areolar left breast (BI-RADS 3). At mammography, no significant findings were found in both breasts.

Core needle biopsy of the enlarged lymph node and of the hypoechoic retro-areolar lesion was performed.

A metallic circular-shape marker was released in breast biopsy site.

No cancer cells were demonstrated at histology in the breast samples (B1, normal breast parenchyma with slight increase in stromal fibrosis), and metastasis of carcinoma no special type (NST) was found in the lymph node (ER 95%, PGR 90%, Ki-67 60%, HER-2 3+).

CUPAx was then suspected and breast MR was performed, according to international guidelines [4,6,9,10].

Breast MR showed a small non-mass enhancement area in the superior pre-pectoral region (BI-RADS 4). No correlation was found at second-look ultrasound.

Therefore, MR-guided breast biopsy was mandatory.

The mammography system used in Mater Olbia Hospital is the Senographe Pristina platform, integrated with SenoBright HD software (GE Healthcare); therefore, taking account of its rapid availability and relative ease-of-use, and according to our initial experience, we decided to perform CESM.

The patient gave written informed consent and was prepared for the examination.

A catheter was placed into the upper arm vein contralateral to the affected breast and the intravenous contrast agent was injected.

Non-ionic contrast agent Iohexol (Omnipaque containing 350 mgI/mL of iodine, GE Healthcare) at a dose of 1.5 mL/Kg at a flow rate of 3 mL/s was used.

Approximately 2 min after injection, the radiographer positioned the patient as required for standard mammography examinations, in the following order: the craniocaudal (CC) projection of the unaffected side, the CC projection of the affected side, the mediolateral oblique (MLO) projection of the affected side, and finally the MLO projections of the unaffected side. The radiographic process was completed in 7 min.

The device in the CESM mode automatically performed a low-energy and a high-energy exposure in each view. A combination of low-energy and high-energy images through specific image processing software was performed to generate subtracted images in each view (with contrast agent uptake information).

CESM showed the presence of a highly suspicious enhancement area with indistinct margins of 15 mm in the superior pre-pectoral region of the left breast, corresponding to the MR-detected lesion.

**Figure 1:**
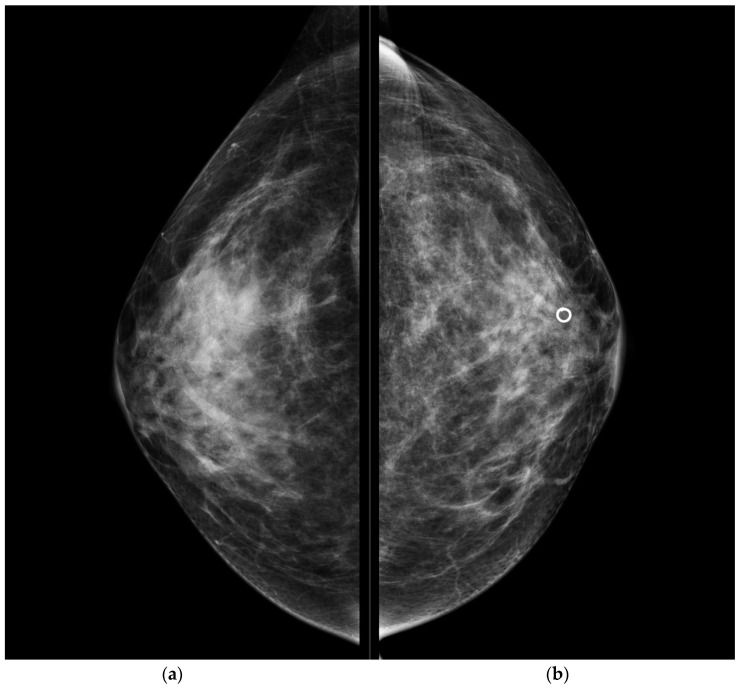
Conventional CC projections of the right breast (**a**) and left breast (**b**). A circular-shape marker released after US-guided biopsy was seen in the retro-areolar left breast. No suspicious mammographic findings were detected.

**Figure 2:**
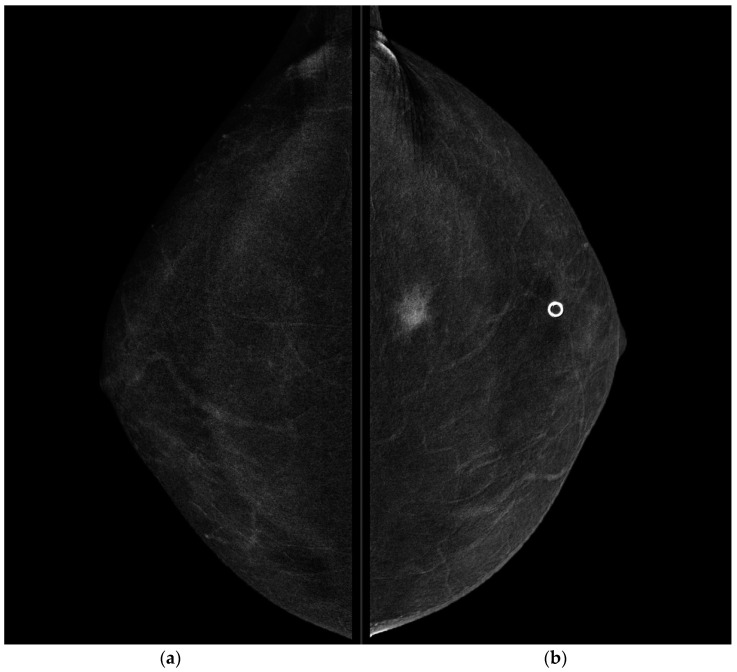
CESM CC projections of the right breast (**a**) and left breast (**b**). Highly suspicious enhancement area with indistinct margins of 15 mm was detected in the pre-pectoral region of the left breast.

**Figure 3:**
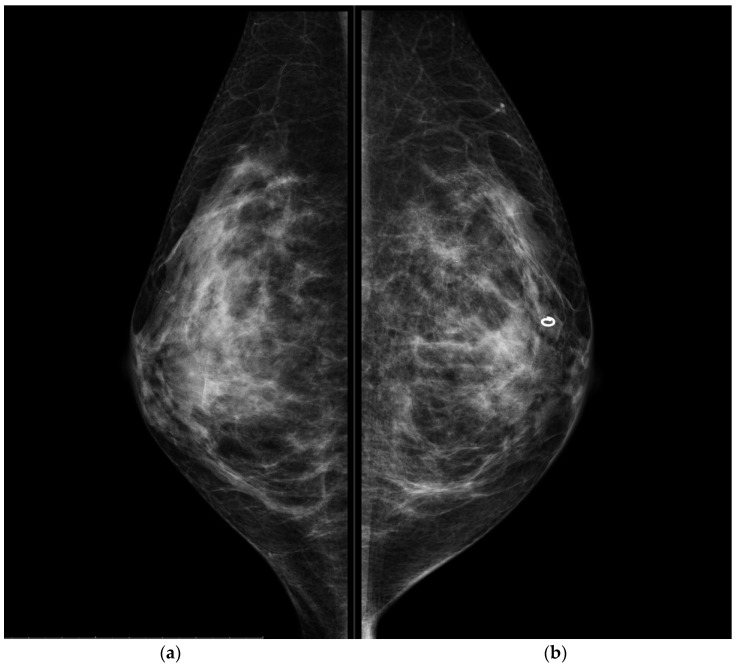
Conventional MLO projections of the right breast (**a**) and left breast (**b**). The circular-shape marker released after US-guided biopsy was present in the retro-areolar left breast. MLO projections, as CC, showed no mammographic alterations.

**Figure 4:**
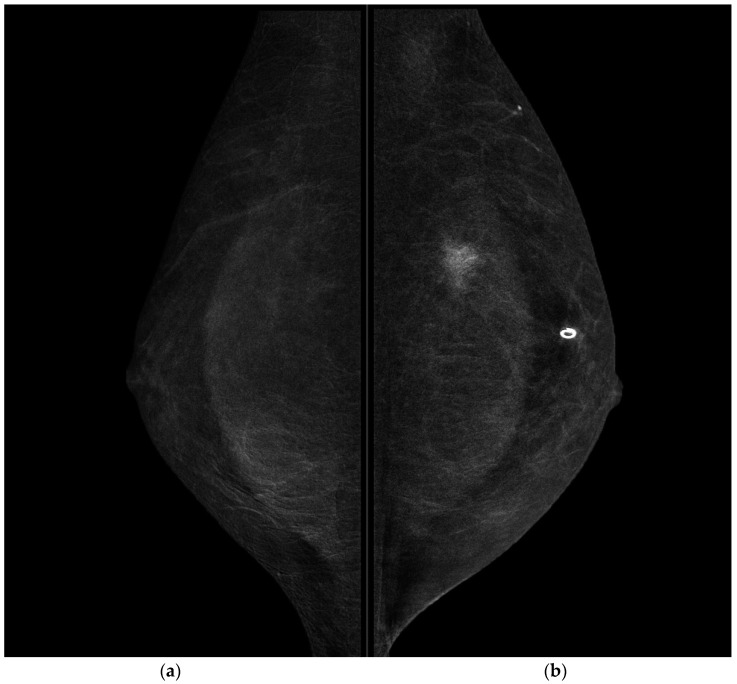
CESM MLO projections of the right breast (**a**) and left breast (**b**). A highly suspicious enhancement area with indistinct margins of 15 mm in the superior pre-pectoral region of the left breast was confirmed in MLO projection.

CESM also confirmed the presence of a metallic marker in the left retro-areolar breast, previously released in the breast biopsy site (Figure 1, Figure 2, Figure 3 and Figure 4).

**Figure 5:**
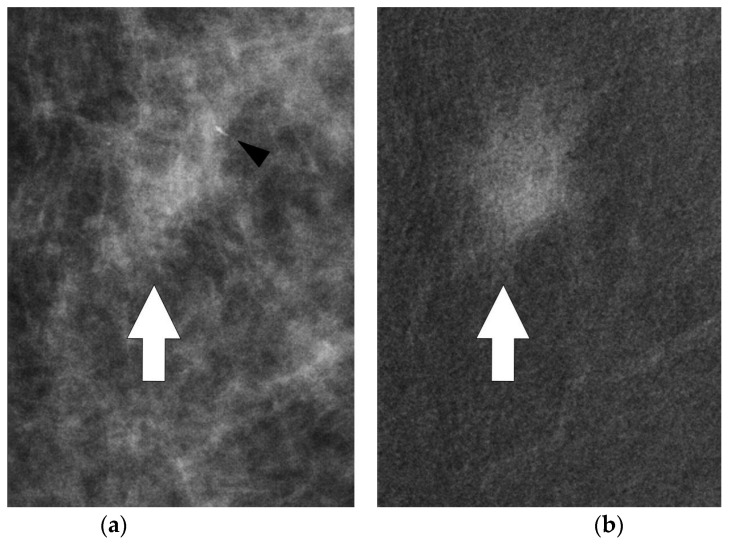
Mammographic magnifications of conventional (**a**) and CESM (**b**) CC projections of the left breast. Some microcalcifications were seen in the anterior margin of the enhancing lesion (black arrowhead). Thick white arrows point to tumor site (**a**,**b**).

**Figure 6:**
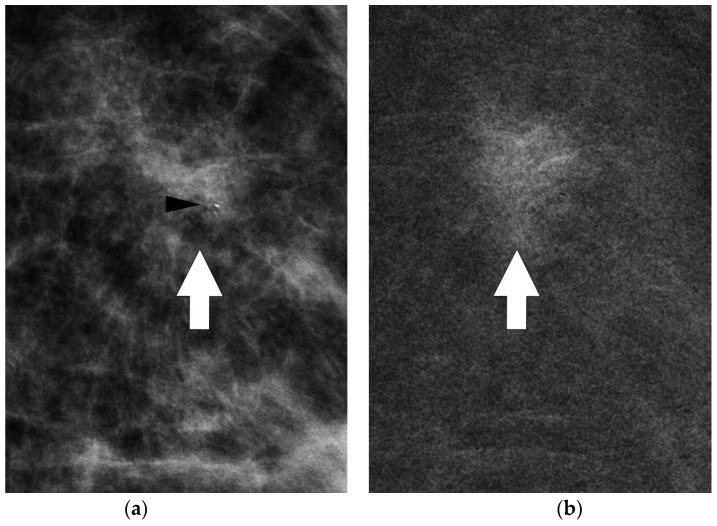
Mammographic magnifications of conventional (**a**) and CESM (**b**) MLO projections of the left breast. Thick white arrows point to tumor site (**a**,**b**). Microcalcifications were confirmed within the enhancing lesion (black arrowhead), allowing a mammographically guided biopsy approach. The combination of mammographic findings and the perfusion pattern improve the detection of minimal signs. Therefore, minimal signs not relevant at conventional mammography become significant after contrast medium administration and can be a target for stereotactic guided biopsy.

Some microcalcifications were seen in the anterior margin of the lesion both in axial and medio-lateral oblique projections, allowing a mammographically guided biopsy approach (Figure 5 and Figure 6).

Stereotactic vacuum-assisted breast biopsy was then performed.

Histological analysis diagnosed a high grade ductal carcinoma in situ (DCIS) with foci of microinvasion (<1 mm).

Hence, MR-guided breast biopsy was avoided, reducing time, costs and human resources.

Following a breast multidisciplinary evaluation (among surgeons, radiologists, oncologists, radiotherapists, pathologists, geneticists and psychotherapists), breast-conserving surgery and axillary dissection were programmed.

The final pathology report was invasive ductal carcinoma (ICD), G3, ER 80%, PGR 80%, Ki-67 45%, HER-2 3+, with negative surgical margins.

## 3. Discussion

Women with axillary lymph node metastases represent a rare cohort of CUP syndrome patients.

If histological analysis of axillary lymph node metastasis suggests an adenocarcinoma or a poorly differentiated carcinoma, the ipsilateral breast is the most obvious site of a primary tumor, especially when immunohistochemistry detects the presence of estrogen or progesterone receptors or HER2 overexpression. In most cases, occult breast cancer is an invasive ductal carcinoma, more rarely an invasive lobular carcinoma [4,8,10,14].

Radiology has a crucial role in the management of CUP syndrome patients and a close collaboration between radiologists and pathologists optimizes the possibility of reaching a specific diagnosis [10].

In the case of patients with probable occult breast cancer, a dedicated breast radiologist promotes the correct diagnostic strategies to identify the cancer.

According to the main guidelines, the first breast imaging step is represented by mammography and breast ultrasound evaluation [10,15,16]. In the case of negative results, it is mandatory to perform second-level breast investigation. Breast MR allows the detection of a breast lesion in more than 50% of occult breast cancer cases [18,19].

As breast-MR specificity is not optimal, any detected lesion must be histologically confirmed [18,19,20]. In the case of a suspicious enhancing lesion at breast MR, a second-look ultrasound and a careful mammography revision must be performed. The identification of an ultrasound alteration in the site of the enhancing lesion could avoid an MR-guided biopsy and allow the performance of a US-guided biopsy [20,23].

Breast MR-guided biopsy is an interventional practice not available in all centers that requires specific expertise, more resources, a long time and the administration of i.v. contrast medium. This biopsy procedure also has intrinsic limits such as the difficulty in accessing very deep or very superficial lesions and the difficulty of working in breasts with reduced thickness [20].

Instead, ultrasound-guided biopsy is a commonly used, fast, simple and inexpensive method. Therefore, the second-look US assumes an increasingly prominent role in order to identify the lesion highlighted by the resonance and then to proceed with the US-guided biopsy [24,25].

Currently, the detection rate of the second-look US has an average value of 57%. This value may be partially justified by the difficulty of translating the information obtained at MR, in which the breast is in prone position, in that obtained at ultrasound evaluation, in which the breast is in supine position. The transition from the prone to the supine position determines changes in the overall thickness of the breast and spatial depiction of the lesion; this displacement can be more or less important and can occur in the same quadrant or in different quadrants. However, small non-mass enhancement lesions at MR are difficult to perceive at ultrasound and the correlation between the MR findings and the ultrasound alterations is not always perfect [21,23].

The MR lesion that is not evident at second-look ultrasound is named the MR-only lesion. In the absence of a clear and evident ultrasound target, the MR-only lesion is typed by MR-guided biopsy [20].

CESM is a new breast imaging technique that combines conventional mammography with the intravenous administration of an iodinated contrast material. Similar to breast MR examination, CESM provides both morphological and functional information [22,26]. Several studies demonstrated the good diagnostic performance of CESM in detecting breast cancer. CESM may be useful for indications previously reserved for MR, and its role in different clinical settings is being assessed [21,27].

CESM offers new diagnostic opportunities; however, further experience and studies with larger case series are needed to confirm its role.

In our preliminary experience and according to current literature [21,27], CESM could accurately help diagnosis in different clinical settings, particularly in patients with MR-contraindications or physical limitations.

Hence, in the time of Precision Medicine, CESM could be identified as a valid alterative to breast MR in patients that need to undertake second-level breast diagnostic exams.

In the presented case, the use of CESM allowed the identification of the lesion at mammogram, avoiding the MR-guided biopsy. The combination of mammographic findings and the perfusion pattern improved the detection of minimal signs. Therefore, minimal signs not relevant at conventional mammography become significant after contrast medium administration and can be a target for stereotactic guided biopsy (Figure 5 and Figure 6).

The use of CESM in CUPAx patients could be helpful in the detection of occult breast cancer in a mammographic setting, reducing the time of diagnosis and the cost of other diagnostic and interventional procedures such as breast MR and MR-guided breast biopsy.

In the case of the identification of primary breast cancer, therapeutic surgical strategy focuses on two fronts, the axillary site and the breast site. Breast conservation or mastectomy and dissection of the first and second lymph node levels are the standard surgical treatment [28,29,30,31].

In the case of no detectable primary breast tumor, the possible therapeutic option in the ipsilateral breast is represented by definitive locoregional treatment (mastectomy or breast conserving surgery), which improved the outcome more than observation [32,33].

Adjuvant chemotherapy and hormone therapy follow the guidelines for patients with stage 2 breast cancer. Survival and prognosis rates are also comparable to those of patients with stage 2 breast cancer. The number of axillary lymph nodes involved and the hormone receptor status are considered significant prognostic predictors [34,35].

## 4. Conclusions

CUP syndrome is an aggressive disease with poor prognosis.

Patients in the favorable subset, since the site of the primary site is likely assumed or detected, have a better treatment and a superior outcome.

CUPAx is a rare condition that is part of the favorable subset of CUP syndrome patients.

The first step in the diagnostic work-up of CUPAx patients is the biopsy of the lymph node metastasis and the execution of first-level breast diagnostic imaging (mammography and ultrasound).

In the case of occult breast cancer, breast MR must be performed. If a lesion is detected at breast MR, a second-look ultrasound is recommended. US-guided or MR-guided biopsy is subsequently performed.

The identification of the primitive tumor improves the possibilities of treatment and prognosis.

Recently, a new breast imaging technique, contrast-enhanced spectral mammography, shows potential in the identification of occult breast cancer in the case of CUPAx.

Several studies and larger experience in clinical practice are necessary to evaluate the role of CESM in this setting of patients.

## Data Availability

Data are contained within the article.
